# Generation of vectorial generalized vortex array with metasurfaces

**DOI:** 10.1038/s41377-025-02102-7

**Published:** 2026-01-22

**Authors:** Qingsong Yao, Zile Li, Guoxing Zheng

**Affiliations:** 1 Electronic Information School, Wuhan University, Wuhan, China; 2 Peng Cheng Laboratory, Shenzhen, China; 3 Wuhan Institute of Quantum Technology, Wuhan, China

**Keywords:** Metamaterials, Nanophotonics and plasmonics

## Abstract

The ability to create complex three-dimensional structures of light is extremely challenging. Now, a technique combining Dammann optimization with metasurfaces has been developed, enabling control over all parameters, including polarization, phase, angular momentum, and spatial modes. The generation of three-dimensional generalized vortex beams can open new horizons for their applications in photonics.

Optical metasurfaces, as artificially engineered subwavelength nanostructure arrays, possess rich design degrees of freedom (DOF), offering a versatile platform for wavefront modulation^1–3^. In particular, compared to conventional bulky optical elements or diffractive optical elements, the arbitrarily tunable anisotropy of metasurfaces has attracted rapidly growing research interest in highly integrated and powerful polarization-control devices. These devices enable diverse functionalities such as polarization beam splitters^4,5^, vectorial wavefront detection^6^, polarization imaging^7^, polarization spectroscopy^8^, nanoprinting^9^ and vector holography^10–12^. Furthermore, more complex vectorial patterns can be developed by encoding multidimensional spin angular momentum (SAM) and orbital angular momentum (OAM) states within a vector field.

In 1992, Allen and his team demonstrated that light beams with helical phase fronts ${e}^{il\theta }$ carry an OAM of $l$ per photon^13^. This came as a surprise, because the OAM provided one of the most important modal bases in structured light and has led to fundamental studies and diverse applications, ranging from communications and optical tweezers to sensing and quantum information processing^14^. A typical example of encoding the SAM and OAM parameters is the vectorial vortex beam (VVB), which carries the total angular momentum of light^15–18^. Currently, the shaping of VVB has extended from two-dimensional (2D) to three-dimensional (3D) space^19^. The generation of VVBs from metasurfaces has been extensively studied and applied in optical encryption, optical computing, optical communications, and optical tweezers and trapping^20,21^. However, most of these modulations are limited to traditional vortex beams with a constant phase gradient, and the investigation of spatially variant vectorial generalized vortex arrays remains unexplored.

In a recent paper published in Light: Science & Applications, Zhang et al. report a significant advance in the generation of a 3D generalized vortex beam (GVB) array with customized amplitude, phase, polarization, and spatial modes from Dammann vortex metasurfaces (DVM)^22^. They build upon the work that the same group reported a few years ago, in which GVBs with on-demand intensity profiles were demonstrated with metasurfaces for the first time (see also Ref. ^23^ for prior work on GVBs generated from metasurfaces). In ordinary optical vortices, the azimuthal phase varies uniformly, so that the profile structure features a donut shape in the transverse plane. In GVBs, the optical phase of the electromagnetic field, instead, is a pre-designed function along the azimuthal direction. As such, this phase variation enables the generation of arbitrary closed-loop profile for the shape of the vortex beam, including polygons, stars, and windmills, by tailoring the local phase gradient along the azimuthal direction. Furthermore, they explored an approach for generating GVB arrays using a diffraction-multiplexed DVM, which provides an opportunity to realize patterns in different diffraction orders for implementing adder and subtractor optical computing^24^.

What Zhang et al. have now added is a further stage of optical manipulation of the vectorial GVBs using a DVM, enabling enhanced control over the vectorial properties and 3D diffraction patterns to increase information capacity. In this manipulation, vectorial diffraction orders with arbitrary beam patterns are first achieved. As shown in Fig. 1, the customized closed-loop beam patterns with diverse polarization are generated in the XYZ space while linearly polarized beams illuminate the DVM. In principle, the phase of the DVM is obtained by judiciously combining the optimized phase profiles of the vortex array metasurface and the Dammann zone plate. Here, the proposed metasurface is composed of nanofins with rectangular cross-sections, enabling independent phase modulation for orthogonal polarization channels. Finally, a set of metasurfaces were fabricated and used in experiments to generate full parameter-modulated 3D GVB arrays.

**Fig. 1:**
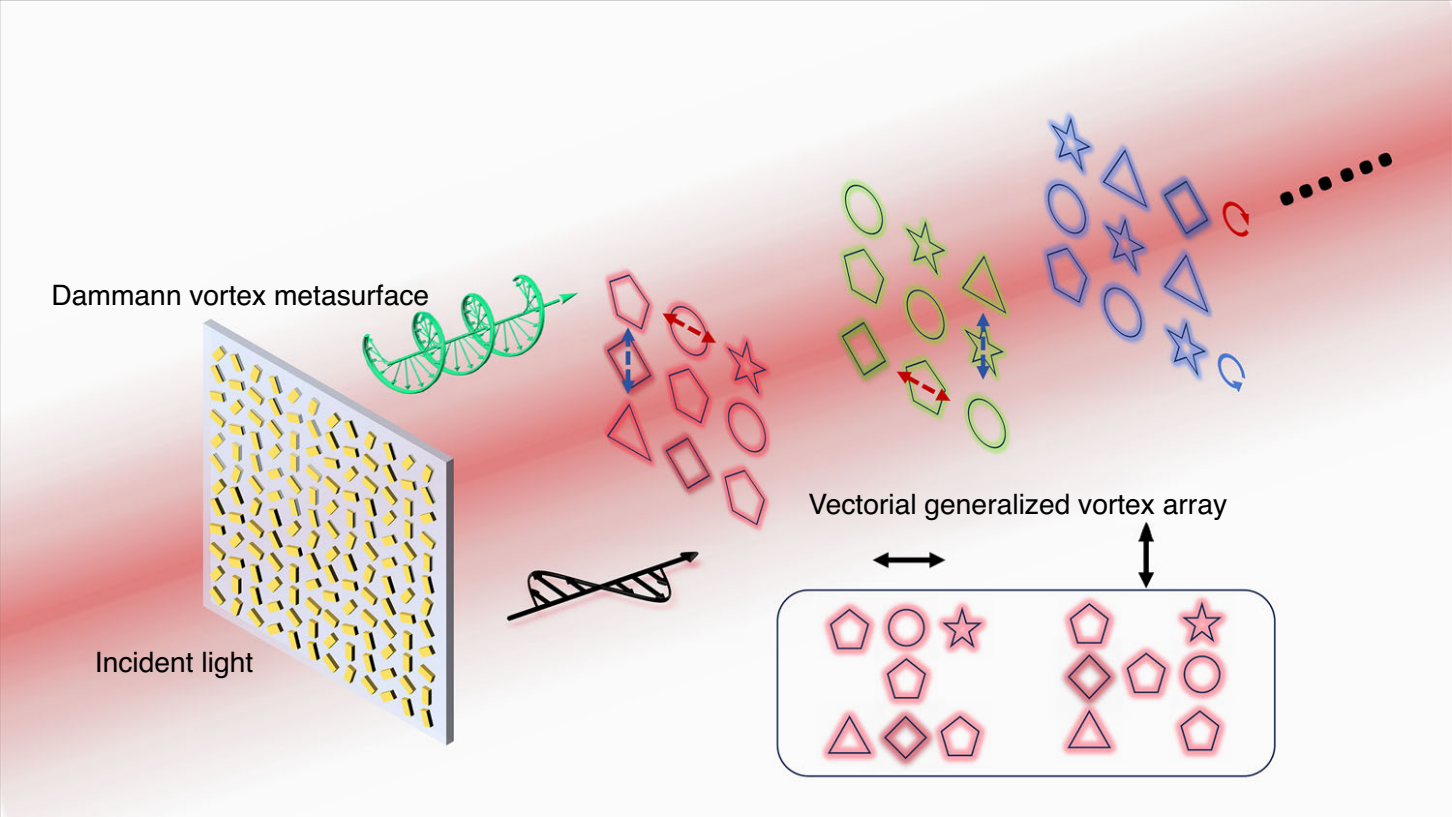
Schematics of the proposed Dammann vortex metasurface for generating a vectorial generalized vortex beam array in 3D space. Under normal incidence, the 3D GVB arrays can be observed at different predefined distances

In summary, this work leverages 2D DOFs offered by metasurfaces, along with a developed phase optimization algorithm, making a remarkable step forward in the comprehensive modulation of lightfield parameters, including amplitude, phase, polarization in 3D spaces. Looking ahead, the development of more powerful metasurface techniques capable of decoupling optical parameters in the Jones matrix^25,26^, along with multifunctional metasurfaces for the independent control of light’s DOF^27,28^, is expected to enable even more sophisticated control over vectorial light fields. For example, the amplitude distribution would no longer be restricted to one-dimensional closed curves, and the polarization within the pattern need not be uniform; instead, it may become possible to realize vector holograms with arbitrarily varying polarization, amplitude and phase. It is anticipated that these technological advances may enable a range of applications, such as optical imaging, optical communication, optical encryption, and optical computing.
